# Supplementary material for: Large ultrafast-modulated Voigt effect in noncollinear antiferromagnet Mn3Sn
Source: Nat Commun. 2021 Sep 6;12:5266. doi: 10.1038/s41467-021-25654-9 (PMC8421456; doi:10.1038/s41467-021-25654-9)
Supplement: Supplementary file 1 — Supplementary Information [file 41467_2021_25654_MOESM1_ESM.pdf]

Supplementary Information for "Large Ultrafast-Modulated Voigt Effect in Noncollinear Antiferromagnet Mn_3Sn"

H. C. Zhao[1], H. Xia[1,2], S. Hu[3], Y. Y. Lv[3], Z. R. Zhao[1], J. He[1], E. Liang[1], G. Ni[1,*], X. P. Qiu[3,*], S. M. Zhou[3,*], L. Y. Chen[1], H. B. Zhao[1,4*]

1 Key Laboratory of Micro and Nano Photonic Structures (Ministry of Education), and Shanghai Ultra-precision Optical Manufacturing Engineering Research Center, Department of Optical Science and Engineering, *Fudan University*, Shanghai, 200433, China

2 Department of Physics, *Fudan University*, Shanghai, 200433, China

3 Shanghai Key Laboratory of Special Artificial Microstructure Materials and Technology and Pohl Institute of Solid State Physics and School of Physics Science and Engineering, *Tongji University*, Shanghai 200092, China

4 Shanghai Frontier Base of Intelligent Optoelectronics and Perception, Institute of Optoelectronics, *Fudan University*, Shanghai, 200433, China

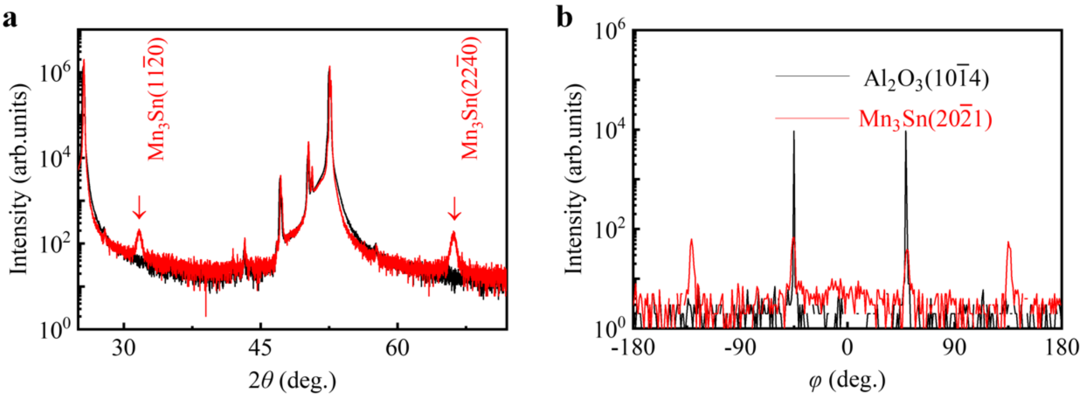

Supplementary Figure 1 | X-ray diffraction (XRD) measurements. (a) The out-of-plane X-ray θ-2θ scan pattern at wide 2θ range for the Mn_3Sn and Al_2O_3 substrate, indicating that the 40-nm thick Mn_3Sn film grown on $Al_2O_3(1\bar{1}02)$ substrate is $(11\bar{2}0)$ oriented. Moreover, X-ray φ scan patterns in figure (**b**) show an exact match for $Mn_3Sn(20\bar{2}1)$ and $Al_2O_3(10\bar{1}4)$. These X-ray results provide pertinent evidence for the high quality epitaxial growth of $(11\bar{2}0)$ Mn_3Sn onto $Al_2O_3(1\bar{1}02)$ substrate.

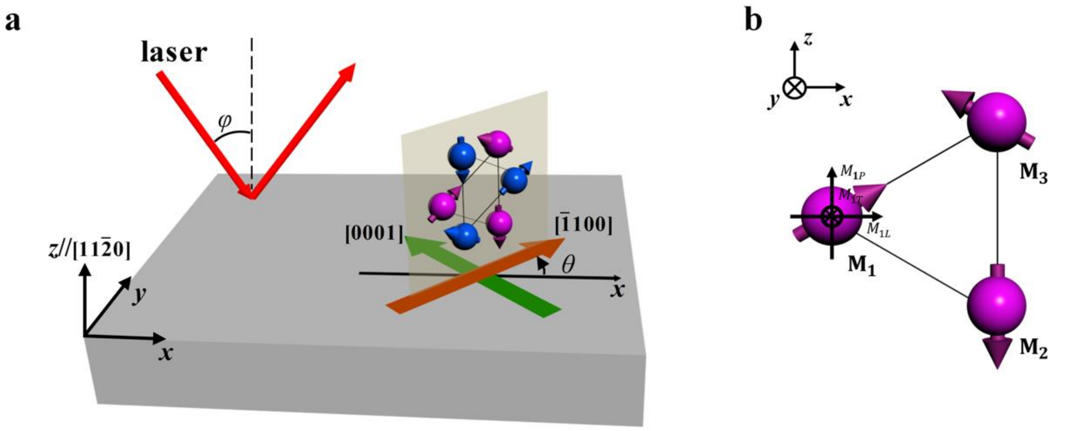

Supplementary Figure 2 |Geometry for simulation of Voigt effect. (a) The right-handed *x*, *y*, *z* coordinate system in Mn_3Sn films. *θ* and *φ* represents the direction of in-plane magnetization and incident light, respectively. Purple and blue spheres with arrows represent Mn spins. (**b)** Mn_3Sn inverse triangular spin structures. $\mathbf{M_1}$, $\mathbf{M_2}$, and $\mathbf{M_3}$ represent the sublattice magnetizations. M_{1L}, M_{1T}, and M_{1P} represent the longitudinal, transverse, and polar components of the sublattice magnetization.

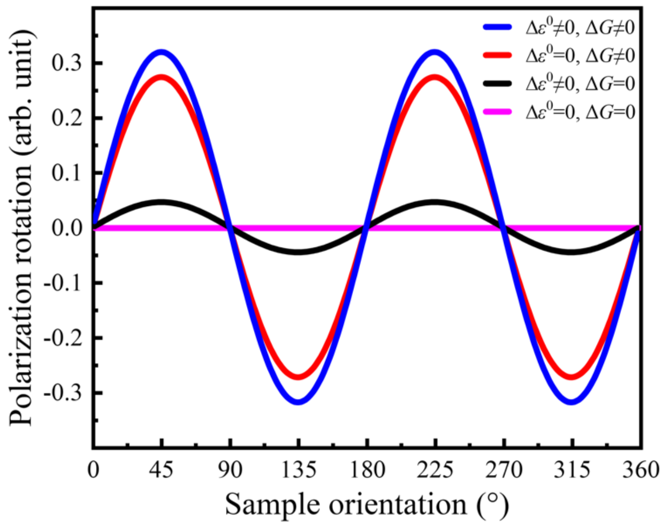

Supplementary Figure 3 |Simulated polarization rotation. Polarization rotation for different permittivity tensor parameters in the non-collinear hexagonal AFM film. Both the Voigt effect and lattice-induced dichroism effect may cause the polarization rotation. $\Delta\varepsilon^0$ and ΔG represent the difference of the zero and quadratic diagonal

permittivity tensor, respectively.

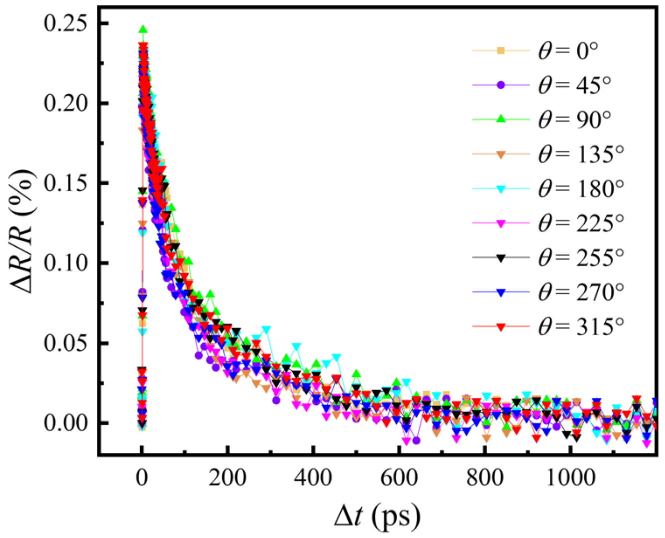

Supplementary Figure 4 | Time-resolved reflectivity measurements. The azimuth dependence of Δ*R* dynamics in Mn_3Sn films. In the main text, we showed the Δ*P* dynamics at various sample orientation *θ* by rotating the sample around its surface normal. For comparison, Supplementary Figure 4 shows the measured Δ*R* dynamics for different sample orientations. In all cases, Δ*R* keeps nearly constant.

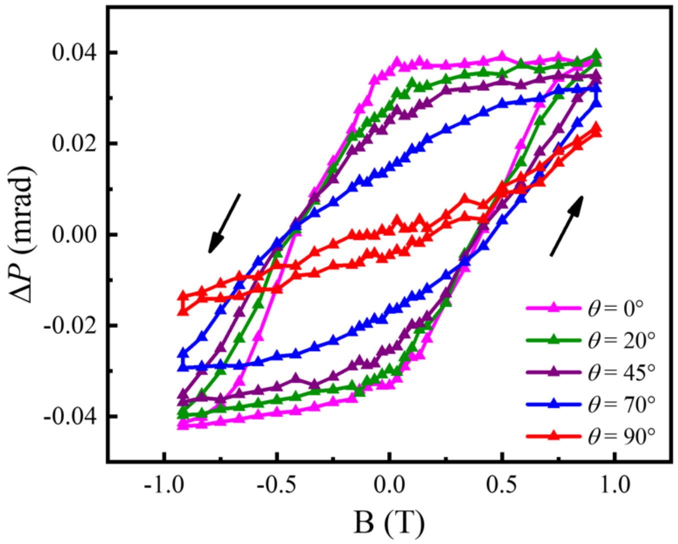

Supplementary Figure 5 | Static MOKE measurements. Static MOKE loops for different sample orientations *θ* measured by a 670-nm semiconductor. Here, the

magnetic field is applied along the *x* axis within the film plane.

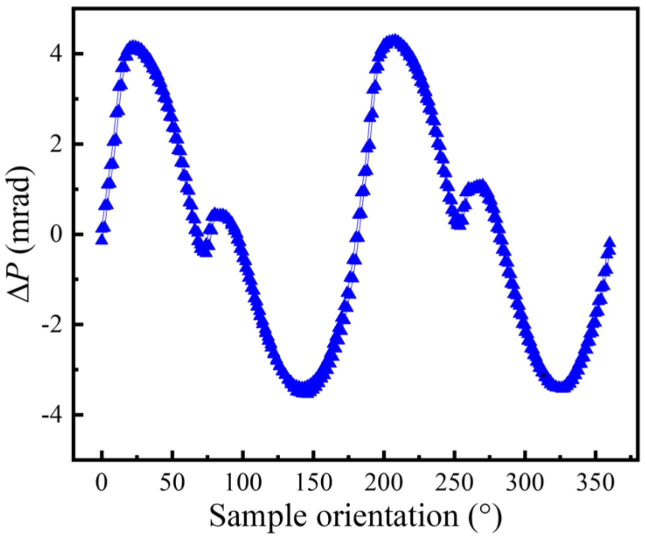

Supplementary Figure 6 | Orientation-dependent static polarization rotation. Sample orientation (θ) dependent polarization rotation ΔP in static measurements by a 670-nm semiconductor laser.

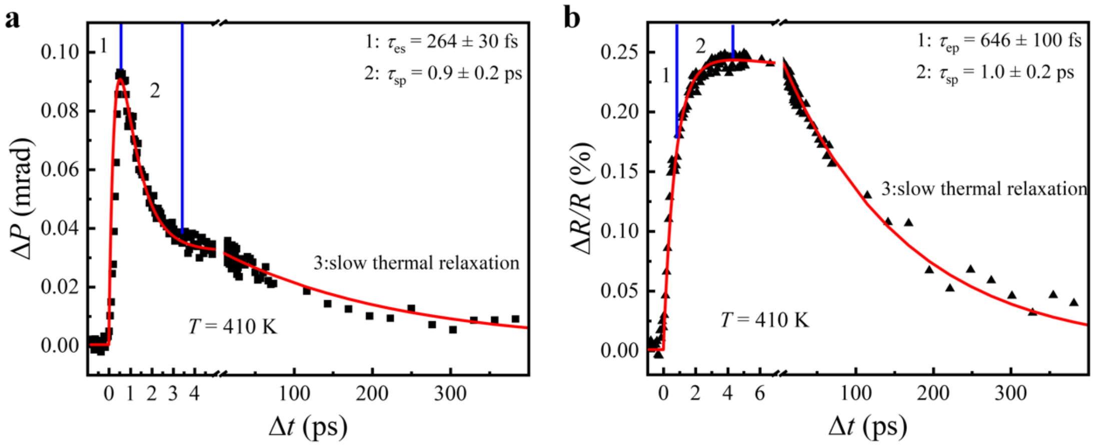

Supplementary Figure 7 | Mn_3Sn ΔP and ΔR dynamics at 410 K. a Dynamic ΔP signals at T = 410 K. **b** Dynamic ΔR signals at T = 410 K. The solid curves correspond to the fitting results. τ_{es}, τ_{ep} and τ_{sp} represent the energy transfer in electronic-spin, electronic-lattice and spin-lattice system, respectively.

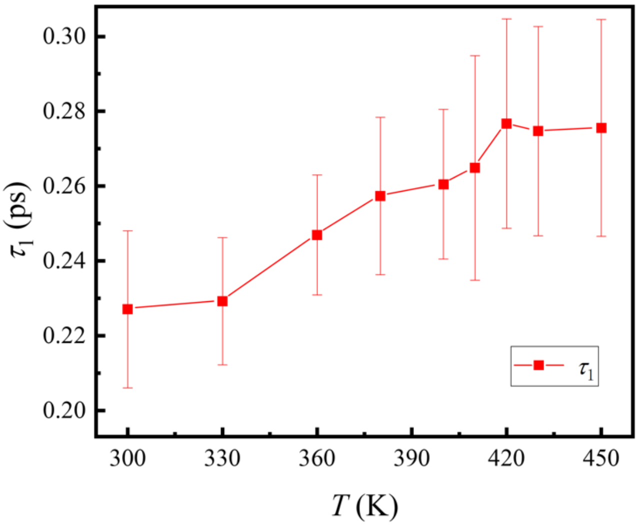

Supplementary Figure 8 | Temperature dependence of the demagnetization time constant τ_1. Error bars correspond to the standard deviation.

Supplementary Note I. Magneto-optical Voigt effect in Mn_3Sn films

For collinear AFM cubic structure, the magneto-optical (MO) Voigt effect has been widely reported [Refs. 12, 13 in the main text], and the polarization rotation due to the MO Voigt effect can be simply described by $\Delta P(\Delta t, \theta) = (2Q/M)\sin 2(\theta - \phi)\, \delta M(\Delta t)$ [Refs. 6, 12 in the main text]. Considering that the spins in non-collinear AFM Mn_3Sn aligns within one plane, analogous to the case of the collinear AFM cubic structure, we have used the above formula to depict the polarization rotation in Mn_3Sn. But for non-collinear AFM Mn_3Sn of hexagonal structure, the strict description of the MO Voigt effect is actually more complicated due to its inverse triangular spin structure and hexagonal lattice. To fully understand the polarization rotation caused by the spin and lattice structure in Mn_3Sn, we analyze its optical response, starting from the Maxwell equations

$$\nabla \times \mathbf{B} = c^{-2} \boldsymbol{\varepsilon} \frac{\partial \mathbf{E}}{\partial t}, \tag{S1a}$$

$$\nabla \times \mathbf{E} = -\frac{\partial \mathbf{B}}{\partial t}, \tag{S1b}$$

where c represents the electromagnetic wave phase velocity in a vacuum and $\boldsymbol{\varepsilon}$ is the

relative permittivity tensor of the medium. Eliminating **B**, the wave equation for the electric field vector is $\nabla^2\mathbf{E} - \nabla(\nabla \cdot \mathbf{E}) = -c^{-2}\boldsymbol{\varepsilon}\frac{\partial^2\mathbf{E}}{\partial t^2}$. Considering the propagation of monochromatic plane wave ($\mathbf{E} = E_0\exp(\mathrm{i}\omega t - \mathrm{i}\mathbf{kr})$) in an anisotropic medium, the wave equation can be written as

$$\begin{pmatrix} \left(\frac{\partial^2}{\partial y^2}+\frac{\partial^2}{\partial z^2}\right) & -\frac{\partial^2}{\partial x\partial y} & -\frac{\partial^2}{\partial x\partial z} \\ -\frac{\partial^2}{\partial y\partial x} & \left(\frac{\partial^2}{\partial z^2}+\frac{\partial^2}{\partial x^2}\right) & -\frac{\partial^2}{\partial y\partial z} \\ -\frac{\partial^2}{\partial z\partial x} & -\frac{\partial^2}{\partial z\partial y} & \left(\frac{\partial^2}{\partial x^2}+\frac{\partial^2}{\partial y^2}\right) \end{pmatrix}\begin{pmatrix} E_x \\ E_y \\ E_z \end{pmatrix} = c^{-2}\begin{pmatrix} \varepsilon_{xx} & \varepsilon_{xy} & \varepsilon_{xz} \\ \varepsilon_{yx} & \varepsilon_{yy} & \varepsilon_{yz} \\ \varepsilon_{zx} & \varepsilon_{zy} & \varepsilon_{zz} \end{pmatrix}\frac{\partial^2}{\partial t^2}\begin{pmatrix} E_x \\ E_y \\ E_z \end{pmatrix}. \quad \text{(S2)}$$

The monochromatic plane wave solutions depend on the permittivity tensor of Mn_3Sn. Here, we establish a right-handed *x*, *y*, *z* coordinate system (shown in Supplementary Fig. 2(a)) with the *z* axis being normal to the sample surface (along Mn_3Sn $[11\bar{2}0]$). The components of the permittivity tensor $\boldsymbol{\varepsilon}$ (3×3 tensor) in a magnetized crystal can be described as

$$\varepsilon_{ij} = \varepsilon_{ij}^{(0)} + K_{ijk}M_k + G_{ijkl}M_kM_l, \quad \text{(S3)}$$

where $\varepsilon_{ij}^{(0)}$ denotes the components of the permittivity tensor when $M = 0$. K_{ijk} and G_{ijkl} are the components of the linear and quadratic magneto-optic tensor. Using the Onsager relation $\varepsilon_{ij}(\mathbf{M}) = \varepsilon_{ji}(-\mathbf{M})$, there have

$$K_{ijk} = -K_{jik}, \quad \text{S(4a)}$$

$$G_{ijkl} = G_{jikl} = G_{ijlk} = G_{jilk}. \quad \text{S(4b)}$$

Using the symmetry operations of the crystal point group, permittivity tensor can be simplified. For the Mn_3Sn hexagonal structure in the coordinate system shown in Supplementary Fig. 2(a), there are eight independent parameters

$$K_{xyz}, K_{zxy}, \quad \text{S(5a)}$$

$$G_{xxxx}, G_{xxzz}, G_{xxyy}, G_{yyxx}, G_{yyyy}, G_{yzyz}. \quad \text{S(5b)}$$

The three magnetic sublattices of the Mn_3Sn triangular order are indicated by the labels M_1, M_2, and M_3 in Supplementary Fig. 2(b). The longitudinal, transverse, and polar components of the sublattice magnetization (e.g., M_{1L}, M_{1T}, and M_{1P}) are defined along the x, y, and z axes, respectively. Taking equation (S5) into equation (S3), the permittivity tensor in Mn_3Sn are

$$\varepsilon_{xx} = \varepsilon_{xx}^{(0)} + G_{xxxx}\left(M_{1L}^2 + M_{2L}^2 + M_{3L}^2\right) + G_{xxyy}\left(M_{1T}^2 + M_{2T}^2 + M_{3T}^2\right) + G_{xxzz}\left(M_{1P}^2 + M_{2P}^2 + M_{3P}^2\right), \qquad \text{S(6a)}$$

$$\varepsilon_{yy} = \varepsilon_{yy}^{(0)} + G_{yyxx}\left(M_{1L}^2 + M_{2L}^2 + M_{3L}^2\right) + G_{yyyy}\left(M_{1T}^2 + M_{2T}^2 + M_{3T}^2\right) + G_{yyxx}\left(M_{1P}^2 + M_{2P}^2 + M_{3P}^2\right), \qquad \text{S(6b)}$$

$$\varepsilon_{zz} = \varepsilon_{xx}^{(0)} + G_{xxzz}\left(M_{1L}^2 + M_{2L}^2 + M_{3L}^2\right) + G_{xxyy}\left(M_{1T}^2 + M_{2T}^2 + M_{3T}^2\right) + G_{xxxx}\left(M_{1P}^2 + M_{2P}^2 + M_{3P}^2\right). \qquad \text{S(6c)}$$

Equation S(6) is obtained for the case where the Mn_3Sn $[\bar{1}100]$ direction is parallel to the x axis. For this case, the symmetrical off-diagonal elements of the permittivity tensor are zero. When the sample are rotated around the sample surface normal, the x-y off-diagonal elements appear. The rotation angle θ (as denoted in Supplementary Fig. 2(a)), is the angle between the Mn_3Sn $[\bar{1}100]$ direction and the x axis of the coordinate system. Using coordinate rotation transform, we obtain the symmetrical off-diagonal elements

$$\varepsilon_{xy} = \varepsilon_{yx} = \frac{1}{2}(\varepsilon_{xx} - \varepsilon_{yy})\sin 2\theta. \qquad \text{S(7)}$$

Using the permittivity tensor in Mn_3Sn, we obtain from equation S(2) two propagating plane wave solutions (E_{xm}, E_{ym}, E_{zm}) with the wavevector k_m. We consider in an isotropic medium a plane wave propagating with an index of refraction n^0 towards the interface at an arbitrary angle φ. The plane of wave incidence is parallel to the xz plane. The wavevector $k_m = \frac{\omega}{c}(\mathbf{x}N_x + \mathbf{z}N_{zm})$, where $N_x = n^0 \sin\varphi$ because of the boundary conditions. The electric and magnetic fields of the incident (i), reflected (r), and transmitted (t) waves can be written as

$$E^i = \left[E_s^i \mathbf{y} + E_p^i(\mathbf{x}\cos\varphi - \mathbf{z}\sin\varphi)\right]\exp(\mathrm{i}\omega t - \mathrm{i}k^i \mathbf{r}), \qquad \text{S(8a)}$$

$$E^r = \left[E_s^r \mathbf{y} + E_p^r(\mathbf{x}\cos\varphi + \mathbf{z}\sin\varphi)\right]\exp(\mathrm{i}\omega t - \mathrm{i}k^r \mathbf{r}), \qquad \text{S(8b)}$$

$$E^t = \sum\nolimits_{m=1,2} \left[E_{xm}\mathbf{x} + E_{ym}\mathbf{y} + E_{zm}\mathbf{z}\right]\exp(\mathrm{i}\omega t - \mathrm{i}k_m\mathbf{r}), \tag{S(8c)}$$

$$H^i = n^0\left[E_p^i\mathbf{y} - E_s^i(\mathbf{x}\cos\varphi - \mathbf{z}\sin\varphi)\right]\exp(\mathrm{i}\omega t - \mathrm{i}k^i\mathbf{r}), \tag{S(8d)}$$

$$H^r = n^0\left[-E_p^r\mathbf{y} + E_s^r(\mathbf{x}\cos\varphi + \mathbf{z}\sin\varphi)\right]\exp(\mathrm{i}\omega t - \mathrm{i}k^r\mathbf{r}), \tag{S(8e)}$$

$$H^t = \sum\nolimits_{m=1,2} \left[-N_{zm}E_{ym}\mathbf{x} - (N_xE_{zm} - N_{zm}E_{xm})\mathbf{y} + N_xE_{ym}\mathbf{z}\right]\exp(\mathrm{i}\omega t - \mathrm{i}k_m\mathbf{r}). \tag{S(8f)}$$

The continuity for the electric and magnetic field components parallel to the interface plane require four equations

$$E_{y1} + E_{y2} = E_s^i + E_s^r, \tag{S(9a)}$$

$$N_{z1}E_{y1} + N_{z2}E_{y2} = n^0\cos\varphi E_s^i - n^0\cos\varphi E_s^r, \tag{S(9b)}$$

$$E_{x1} + E_{x2} = \cos\varphi E_p^i + \cos\varphi E_p^r, \tag{S(9c)}$$

$$-N_x(E_{z1} + E_{z2}) + N_{z1}E_{x1} + N_{z2}E_{x2} = n^0E_p^i - n^0E_p^r. \tag{S(9d)}$$

The solutions of equation S(9) provide the amplitudes E_p^r and E_s^r in terms of the amplitudes of the incident waves E_p^i and E_s^i. Finally, the angle of polarization rotation is derived by the permittivity tensor

$$\Phi_s = -\frac{r_{ps}}{r_{ss}} = -\frac{E_p^r/E_s^i}{E_s^r/E_s^i}, \tag{S(10a)}$$

$$\Phi_p = \frac{r_{sp}}{r_{pp}} = \frac{E_s^r/E_p^i}{E_p^r/E_p^i}. \tag{S(10b)}$$

Using equations S(1)-S(10), we may calculate the polarization rotation of linearly polarized light reflected from the Mn_3Sn film. If neglecting the difference between $\varepsilon_{xx}^{(0)}$ and $\varepsilon_{yy}^{(0)}$, we obtain the analytical formula identical to Equation (1) presented in the main text for the MO Voigt effect. But no simple analytical expression can be obtained in the case of $\varepsilon_{xx}^{(0)} \neq \varepsilon_{yy}^{(0)}$. We then numerically simulate the optical response and obtain the polarization rotation at an incident angle of 40 degrees for the non-collinear hexagonal AFM Mn_3Sn film. From the simulation, we found that the polarization rotation signals are originated from two factors: 1) $\Delta\varepsilon^0 = \varepsilon_{xx}^{(0)} - \varepsilon_{yy}^{(0)}$,

which induces the linear dichroism effect of the lattice; 2) $\Delta G = G_{xxxx} + G_{xxzz} - 2G_{yyxx}$, which represents the difference of the quadratic diagonal tensor $\varepsilon_{xx}^{(2)}$ and $\varepsilon_{yy}^{(2)}$ and causes the MO Voigt effect. The two effects result in very similar trends of polarization rotation as a function of the sample orientation, as can be seen from the simulated results shown in Supplementary Fig. 3. Actually, the simulated polarization rotation of the MO Voigt effect can be approximately described by Equation (5) in the main text. It is important to point out that the modulated MO Voigt effect gives the dominant contribution to the ultrafast laser induced transient polarization rotation according to its temperature dependence and the comparison with the reflectivity dynamics presented in the main text, although the lattice and magnetic structure lead to the similar orientation dependence of the polarization rotation.

Supplementary Note II. Orientation-dependent static polarization rotation measurements:

We used a 670-nm semiconductor laser to measure the polarization rotation P of the light reflected from the sample by adopting crossed polarizers close to the extinction. We applied the horizontal incident plane with the *s*-polarized laser incident at a small angle. The sample was placed on a motorized rotation stage, allowing the measurement of the polarization rotation as a function of the angle (θ) of the $[\bar{1}100]$ axis with respect to the x axis. Polarization rotation results are shown in Supplementary Fig. 6. In principle, both the Voigt effect of the magnetic structure and the linear dichroism effect of the lattice structure can contribute to the polarization rotation in this static measurement.
